# The Cerebrospinal Fluid Secretion Rate Increases in Awake and Freely Moving Rats but Differs With Experimental Methodology

**DOI:** 10.1002/advs.202412469

**Published:** 2025-03-12

**Authors:** Trine L. Toft‐Bertelsen, Beatriche L. Edelbo, Annette B. Steffensen, Sara D. Lolansen, Jonathan H. Wardman, Dennis B. Jensen, Nanna MacAulay

**Affiliations:** ^1^ Department of Neuroscience University of Copenhagen Blegdamsvej 3 Copenhagen N DK‐2200 Denmark

**Keywords:** acetazolamide, CSF, CSF measurements, direct method, ventriculo‐cisternal perfusion assay

## Abstract

Cerebrospinal fluid (CSF) dynamics hold implications for neurological health. Despite its importance, accurate quantification of the CSF secretion rate remains a challenge due to methodological controversies and the influence of anesthesia. A novel technique is established to determine CSF dynamics in awake and freely moving rats, and the CSF secretion is quantified with three different methodologies. The CSF secretion rate is higher in awake rats than in anesthetized rats, the latter demonstrating no requirement for mechanical ventilation for optimal CSF quantification. The CSF secretion rate is ≈10‐fold lower with the “direct method” than with the ventriculo‐cisternal perfusion assay, although the relative acetazolamide‐mediated reduction in CSF secretion is similar across three tested methods. The findings demonstrate the importance of awake models for optimal quantification of the absolute rate of CSF secretion but highlight the versatility of methodologies for the determination of relative changes in CSF secretion associated with inhibitors, age, sex, and various pathologies.

## Graphical abstract

CSF dynamics are vital for neurological health. Yet accurate quantification of CSF rates is challenging. Using a novel technique in awake, freely moving rats, significantly higher secretion rates are found than in anesthetized rats. The findings highlight the importance of awake models and the adaptability of methods for studying CSF changes influenced by inhibitors, age, sex, and pathologies.

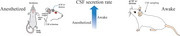

## 1 Introduction

Our brain is bathed in the cerebrospinal fluid (CSF), which is mainly produced by a specialized tissue, the choroid plexus, located in the fluid‐filled brain ventricles. In adult humans, ≈500 mL CSF is secreted daily and circulates through the ventricular system prior to its reabsorption.^[^ ^1^ ^]^ The CSF protects the brain from mechanical insults, serves as the intracerebral dispersion route for signaling molecules and nutrients, and acts as a conduit for waste clearance.^[^ ^2^, ^3^ ^]^ The CSF is thus indispensable for normal brain function. Dysregulation of CSF homeostasis occurs in many pathologies and may lead to debilitating, or even fatal, brain water accumulation represented as the ventriculomegaly signifying hydrocephalus; “water in the head.” Hydrocephalus is the most common entity addressed by neurosurgeons^[^ ^4^ ^]^ accounting for up to 3% of pediatric hospital charges,^[^ ^5^ ^]^ which, taken together with the adult patient group and outpatient care, makes hydrocephalus a global healthcare burden. Hydrocephalus formation has generally been assigned to a blockage of the CSF drainage routes, but emerging evidence suggests that a component of CSF hypersecretion may contribute to the ventriculomegaly underlying some forms of hydrocephalus, e.g. choroid plexus papilloma, choroid plexus hyperplasia, and post‐hemorrhagic hydrocephalus.^[^ ^6^, ^7^, ^8^ ^]^ Most hydrocephalus patients are traditionally treated by invasive neurosurgical intervention, although milder forms of hydrocephalus may be treated with corticosteroids,^[^ ^9^ ^]^ the loop diuretic furosemide, or acetazolamide (Diamox®).^[^ ^10^ ^]^ The latter has been demonstrated to reduce the CSF secretion and thus the intracranial pressure (ICP) in rodents^[^ ^11^ ^]^ and in patients,^[^ ^10^, ^12^, ^13^ ^]^ although with some concerns regarding its effectiveness in treating diseases of elevated ICP,^[^ ^14^, ^15^ ^]^ in part due to associated systemic side effects.^[^ ^16^, ^17^ ^]^ Our limited understanding of the CSF secretion apparatus represents a knowledge gap in our search for a novel efficient and specific therapeutic treatment of elevated ICP.

Despite the century‐long awareness of choroid plexus‐mediated CSF secretion, it has proven challenging for the field to agree on an experimental technique to reliably determine the absolute value of the CSF secretion rate in mammals.^[^ ^18^ ^]^ Particularly challenging are human CSF secretion rates, where modern ethical considerations mostly prevent the invasive methodology employed for the quantitative approaches, which thus prevents robust validation of non‐invasive methods such as magnetic resonance imaging of pulsating aqueductal CSF flow.^[^ ^19^, ^20^ ^]^ The ventriculo‐cisternal perfusion assay, which relies on dilution of a perfused CSF indicator by de novo secreted CSF, has been considered the gold standard for CSF secretion determination in goats, cats, dogs, rats, rabbits, and mice since its introduction in the 1960ies.^[^ ^21^, ^22^ ^]^ More recently, a new so‐called “direct method” was introduced for rodent experimentation, based on blockage of the Aqueduct of Sylvius and quantification of CSF exiting through a brain cannula placed in the lateral ventricle.^[^ ^23^ ^]^ However, the rates of CSF secretion obtained with these two approaches appear to differ by nearly an order of magnitude,^[^ ^23^, ^24^, ^25^, ^26^ ^]^ but these methods remain to be compared in a parallel experimental series, as do their ability to correctly reflect relative changes in CSF secretion rates with inhibitors of choroid plexus transporters involved in CSF secretion, age‐, sex‐, and pathology‐dependent conditions. Notably, the “direct method” does not permit mechanical ventilation of the anesthetized animal, which is predicted to affect the physiological parameters, and hence, indirectly, modulate the CSF secretion rate. Lastly, the anesthesia protocols generally employed to study CSF dynamics^[^ ^11^, ^24^, ^27^ ^]^ may affect the rate of CSF secretion in different ways but remain unresolved due to a lack of CSF secretion rate quantification in awake and freely moving rats.

Here, we present a novel experimental methodology to determine the CSF secretion rate in awake and freely moving rats and reveal the subsequent effect of various anesthesia paradigms on their secretion rate. We quantify the CSF secretion rate with and without mechanical ventilation and with different experimental methodologies, in addition to the relative changes in CSF secretion obtained with a conventional inhibitor of CSF secretion. With a complementary set of methodologies within experimental physiology, conducted with proper surveillance and quantification of related physiological parameters, we here present quantification and validation of various methods, including the novel approach with awake rats, employed to determine CSF secretion rates in experimental rodents.

## 2 Results

### 2.1 Anesthesia Reduces the CSF Secretion Rate

**Figure 1:**
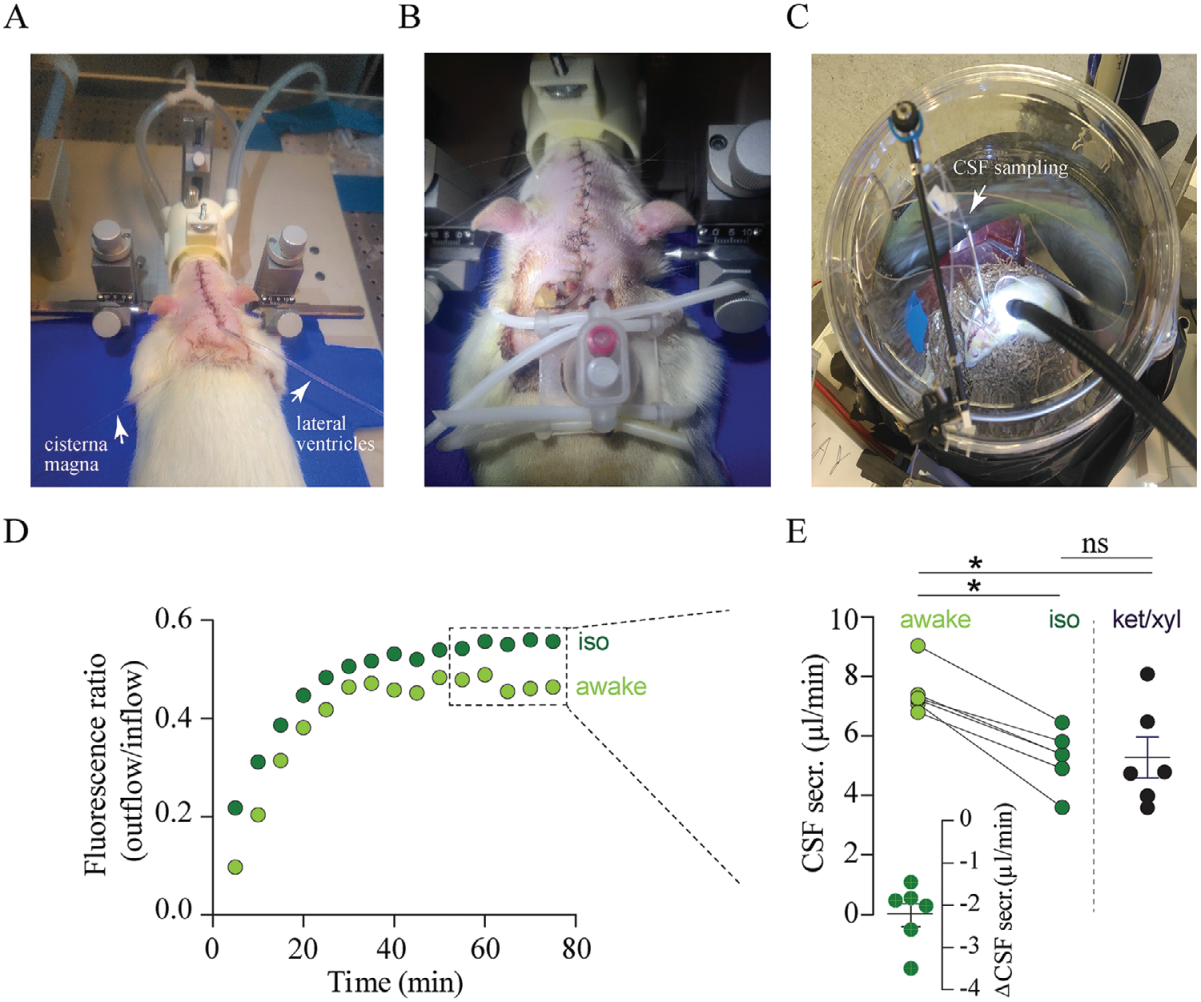
Decreased CSF secretion in anesthetized rats. A) Surgical insertion of brain cannulas secured in the lateral ventricles and cisterna magna of an anesthetized rat. B) Custom‐made tubings attached to a harness and connected to the brain cannulas prior to CSF sampling. C) An awake and freely moving rat roaming the test chamber with a moveable arm securing the tubings in place during the determination of the CSF secretion rate. D) Representative time courses of the fluorescence ratio (outflow/inflow) of dextran during ventriculo‐cisternal perfusion in an awake rat (awake) and under isoflurane anesthesia (iso). E) (left) CSF secretion rates in awake rats (awake) and under isoflurane anesthesia (iso) quantified at time points 55–75 min (indicated by the dashed box in D), n = 6 of each; E (inset): intra‐rat isoflurane‐dependent reduction in CSF secretion; E (right) CSF secretion rates in batch‐matched rats anesthetized with xylazine and ketamine (ket/xyl), n = 6. Statistical significance was determined by one‐way ANOVA with Tukey's *post‐hoc* test. ^*^ *P* < 0.05, ns = non‐significant.

To determine the rate of CSF secretion in awake and freely moving rats, we modified the classic ventriculo‐cisternal perfusion assay to allow CSF secretion rate measurements in non‐anesthetized and freely moving rats. The ventriculo‐cisternal perfusion assay is based on equiosmolar, preheated, gas‐equilibrated HCO_3_^−^‐buffered, dextran‐containing artificial CSF (aCSF) perfusion into the lateral ventricle of a rat with simultaneous CSF sampling from a cisterna magna puncture. The dilution of the perfused dextran occurs with the de novo CSF synthesis and is employed to calculate the CSF secretion rate.^[^ ^25^, ^28^ ^]^ To implant brain access ports to be employed for subsequent ventriculo‐cisternal perfusion in awake rats, we surgically inserted brain cannulas in the lateral ventricle and in cisterna magna of the anesthetized rats (**Figure** **1**A) with the subsequent attachment of custom‐made tubings to a harness worn by the rats (Figure 1B). Following a ≈16 h recovery, the rats were briefly (<10 min) anesthetized with isoflurane to confirm fluid flow before placing the rats in the test chamber with a moveable arm securing the tubes in place (Figure 1C). The perfusion of equiosmolar, preheated, gas‐equilibrated HCO_3_^−^‐buffered, dextran‐containing a CSF by a peristaltic mini pump was initiated following ≈10 min recovery. CSF was continuously sampled from the cisterna magna puncture with another peristaltic mini pump and the CSF secretion rate was calculated from the dilution (fluorescence ratio outflow/inflow) at the time of plateau (Figure 1D). The CSF secretion rate in the awake and freely moving rats was 7.49 ± 0.32 µL min^−1^, n = 6 rats (Figure 1E, left). To determine the influence of isoflurane anesthesia on the rate of CSF secretion, the same rat was subsequently anesthetized with isoflurane, and its CSF secretion rate was determined with the same experimental equipment and tubings as before. To ensure that the order of experimentation did not influence the obtained CSF secretion rates, half of the experiments were conducted as awake‐isoflurane and half as isoflurane‐awake with 90 min intervals (randomized alternate order). Isoflurane anesthesia significantly lowered the CSF secretion rate to 5.25 ± 0.39 µL min^−1^, n = 6 rats, P < 0.05 (Figure 1E, left), with an intra‐rat reduction of 29.8% (2.21 ± 0.30 µL min^−1^, *P* < 0.001, Figure 1E, inset). To compare with another generally employed anesthesia, we determined the CSF secretion rate in separate, but batch‐matched, rats anesthetized with xylazine and ketamine (xyl/ket) and mechanically ventilated to ensure optimal physiological conditions. The CSF secretion rate under xyl/ket anesthesia (5.22 ± 0.69 µL min^−1^, n = 6 rats, Figure 1E, right) was significantly lower than that obtained in awake rats (P < 0.05), but similar to that obtained during isoflurane anesthesia (P = 1.00). Anesthesia of either tested kind thus reduces the CSF secretion rate compared to that obtained in awake and freely moving rats.

### 2.2 Mechanical Ventilation does not Affect the CSF Secretion Rate in Rats and Mice

**Figure 2:**
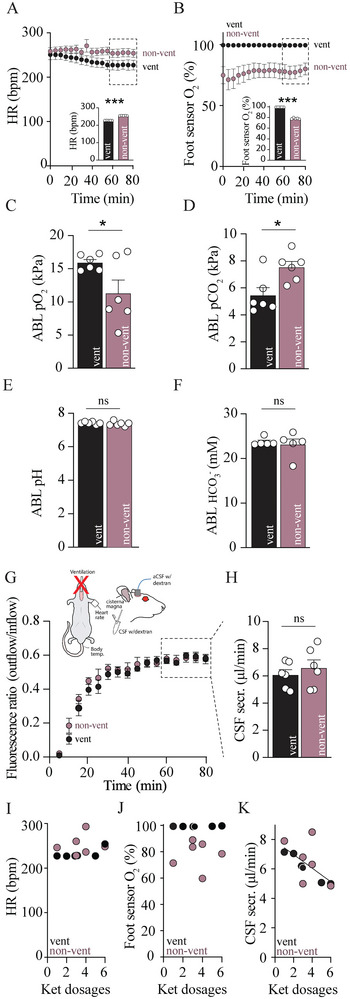
Mechanical ventilation does not affect the CSF secretion rate in rats. A) Time courses of heart rate (HR) and oxygen saturation (O_2_) measured with a foot sensor B) in mechanically ventilated and non‐ventilated rats. Insets: Summarized and averaged heart rate (HR) and oxygen saturation (O_2_) at time point 60–80 min. C) Summarized and averaged partial oxygen pressure (pO_2_), D) carbon dioxide pressure (pCO_2_), E) pH_plasma_ and (F) [HCO_3_^−^]_plasma_ determined from blood gas measurements (ABL) at time point 80 min, n = 6 of each. G) Summarized time courses of the fluorescence ratio (outflow/inflow) of dextran in ventilated and non‐ventilated rats and H) summarized CSF secretion rates at time points 60–80 min (indicated by dashed box in G), n = 6 of each. Inset: schematic of the experimental setup. I) Heart rate (HR), J) oxygen saturation (O_2_) and K) CSF secretion as a function of a number of ketamine re‐dosages, n = 6 of each. Statistical significance was determined by Student's *t*‐test and correlations were determined by simple linear regression. ^*^ *P* < 0.05, ^**^ *P* < 0.01, ns = non‐significant.

**Figure 3:**
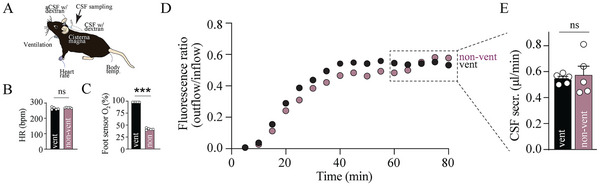
Mechanical ventilation does not affect the CSF secretion rate in mice. A) Schematic of the experimental setup. B,C) Summarized and averaged heart rate (HR) and oxygen saturation (O_2_) as measured with a foot sensor in mechanically ventilated and non‐ventilated mice, n = 5 of each. D) Representative time courses of the fluorescence ratio (outflow/inflow) of dextran during ventriculo‐cisternal perfusion in ventilated and non‐ventilated mice. E) Summarized CSF secretion rates at time point 60–80 min (indicated by the dashed box in D), n = 5 of each. Statistical significance determined by Student's *t*‐test. ^***^ *P* < 0.001, ns = non‐significant.

The anesthesia‐induced reduction of CSF secretion could occur as a result of altered physiological parameters, in part modulated by insufficient oxygenation of the anesthetized animal.^[^ ^25^, ^29^ ^]^ To determine the effect of physiological parameters on the CSF secretion rate in anesthetized rats, we conducted a series of ventriculo‐cisternal perfusion assays with an experimental group receiving standard mechanical ventilation under xyl/ket anesthesia and one without. Notably, three rats in the non‐ventilated experimental group died prior to the completion of the experiment and were omitted from the quantitation of the physiological parameters and CSF secretion rates. The non‐ventilated rats sustained elevated heart rate throughout the experimental duration with (255 ± 1 bpm at time point 60–80 min, n = 5) compared to that of the ventilated rats (227 ± 1 bpm, n = 5 of each, P < 0.001, **Figure** **2**A). This increased heart rate occurred, in part, as a response to the reduced arterial oxygen (O_2_) saturation in the non‐ventilated rats (78.1 ± 0.5% at time point 60–80 min, n = 5) compared to that of the ventilated rats (99.4 ± 0.1%, n = 5, P < 0.001, Figure 2B). Blood gas quantification at the termination of the experiment confirmed the reduced partial oxygen pressure (pO_2_) in non‐ventilated rats (11.3 ± 2.0 kPa, n = 6) compared to those ventilated (15.9 ± 0.5 kPa, n = 6, P < 0.05, Figure 2C), and oppositely for the partial carbon dioxide pressure (pCO_2_) with 7.53 ± 0.43 kPa for non‐ventilated rats versus 5.45 ± 0.58 kPa for ventilated rats, n = 6 of each, P < 0.05, Figure 2D. Nevertheless, the blood pH was undisturbed by lack of mechanical ventilation (7.42 ± 0.03 in ventilated rats vs 7.37 ± 0.06 in non‐ventilated rats, n = 6, P = 0.45, Figure 2E), as was the blood [HCO_3_^−^] (23.7 ± 0.4 mm in ventilated rats vs 23.1 ± 1.3 mm in non‐ventilated rats, n = 5, P = 0.68, Figure 2F), and blood electrolytes (Na^+^, K^+^, Cl^−^) and metabolites (glucose and lactate), Figure S1 (Supporting Information). Despite the altered blood oxygenation in the non‐ventilated rats, their CSF secretion rate (6.62 ± 0.60 µL min^−1^, n = 6) was similar to that obtained in ventilated rats (6.13 ± 0.39 µL min^−1^, n = 6, P = 0.49, Figure 2G,H). Notably, although increasing number of ketamin re‐dosages (required to keep the rat fully anesthetized) did not affect the heart rate (P = 0.36, Figure 2I) or the blood oxygenation (P = 0.95, Figure 2J), whether or not the rats were ventilated, the CSF secretion rate was inversely proportional to the number of ketamine re‐dosages during the experimental duration (0.46 µL min^−1^ per re‐dosage, n = 12, R^2^ = 0.41, P < 0.05, Figure 2K). To extend our finding to mice, we conducted a parallel series of ventriculo‐cisternal perfusion assays with and without mechanical ventilation of these animals (**Figure** **3**A). The heart rate remained stable in both non‐ventilated (269 ± 1 bpm) and ventilated (264 ± 3 bpm, n = 5 of each, P = 0.25) mice (Figure 3B), whereas the arterial O_2_ saturation was significantly reduced in the non‐ventilated (41.7 ± 0.8%) compared to the ventilated (98.0 ± 0.0%, n = 5 of each, P < 0.001) mice (Figure 3C). Despite the altered arterial blood oxygenation in the non‐ventilated mice, their CSF secretion rate (0.57 ± 0.07 µL min^−1^, n = 5) was similar to that obtained in the ventilated mice (0.55 ± 0.02 µL min^−1^, n = 5, P = 0.73, Figure 3D,E). Notably, two of the non‐ventilated mice died toward the end of the experimental procedure.

### 2.3 The CSF Secretion Rate Differs with Methodology but not with Infusion Rate

**Figure 4:**
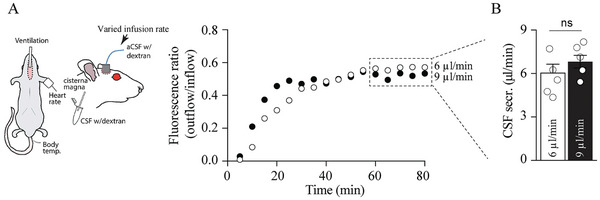
The CSF secretion rate does not differ with infusion rate. A) Schematic of the experimental setup with representative time courses of the fluorescence ratio (outflow/inflow) of dextran during ventriculo‐cisternal perfusion with an infusion rate of 6 or 9 µL min^−1^. B) Summarized CSF secretion rates with 6 or 9 µL min^−1^ infusion rates at time points 60–80 min (indicated by the dashed box in A), n = 5 of each. Statistical significance determined by Student's *t*‐test. ns = non‐significant.

The CSF secretion rate increases with the size of the animal and hence with the size of the brain and the choroid plexus, with an ensuing fairly stable CSF secretion rate per choroid plexus mass.^[^ ^30^ ^]^ Nevertheless, it has proven difficult to define an exact rate of CSF secretion, as the different methodological approaches employed arrive at different values, and/or provide only relative changes with age, sex, inhibitor exposure, pathology, etc.^[^ ^7^, ^11^, ^23^, ^24^, ^25^, ^31^ ^]^ We therefore conducted comparative experimental assessments of the CSF secretion rate with the two most used techniques; the ventriculo‐cisternal perfusion assay and the “direct method”. Prior to comparison of the two methods, we determined whether the rate of infusion of a CSF into the lateral ventricle during a ventriculo‐cisternal perfusion assay affects the CSF secretion rate in anesthetized and ventilated rats. We conducted a set of experiments with our regular perfusion rate of 9 µL min^−1[^ ^28^, ^32^, ^33^ ^]^ compared to a perfusion rate of 6 µL min^−1^. The lower perfusion rate appeared to prolong the time until a stable baseline in the dilution rate was reached (**Figure** **4**A), but with no significant difference between the CSF secretion rates obtained with these two rates (6.01 ± 0.59 µL min^−1^ with an infusion rate of 6 µL min^−1^ and 6.82 ± 0.43 µL min^−1^ with an infusion rate of 9 µL min^−1^, n = 5 of each, P = 0.32, Figure 4B). We therefore employed the infusion rate of 9 µL min^−1^ in the experiments based on the ventriculo‐perfusion assay.

**Figure 5:**
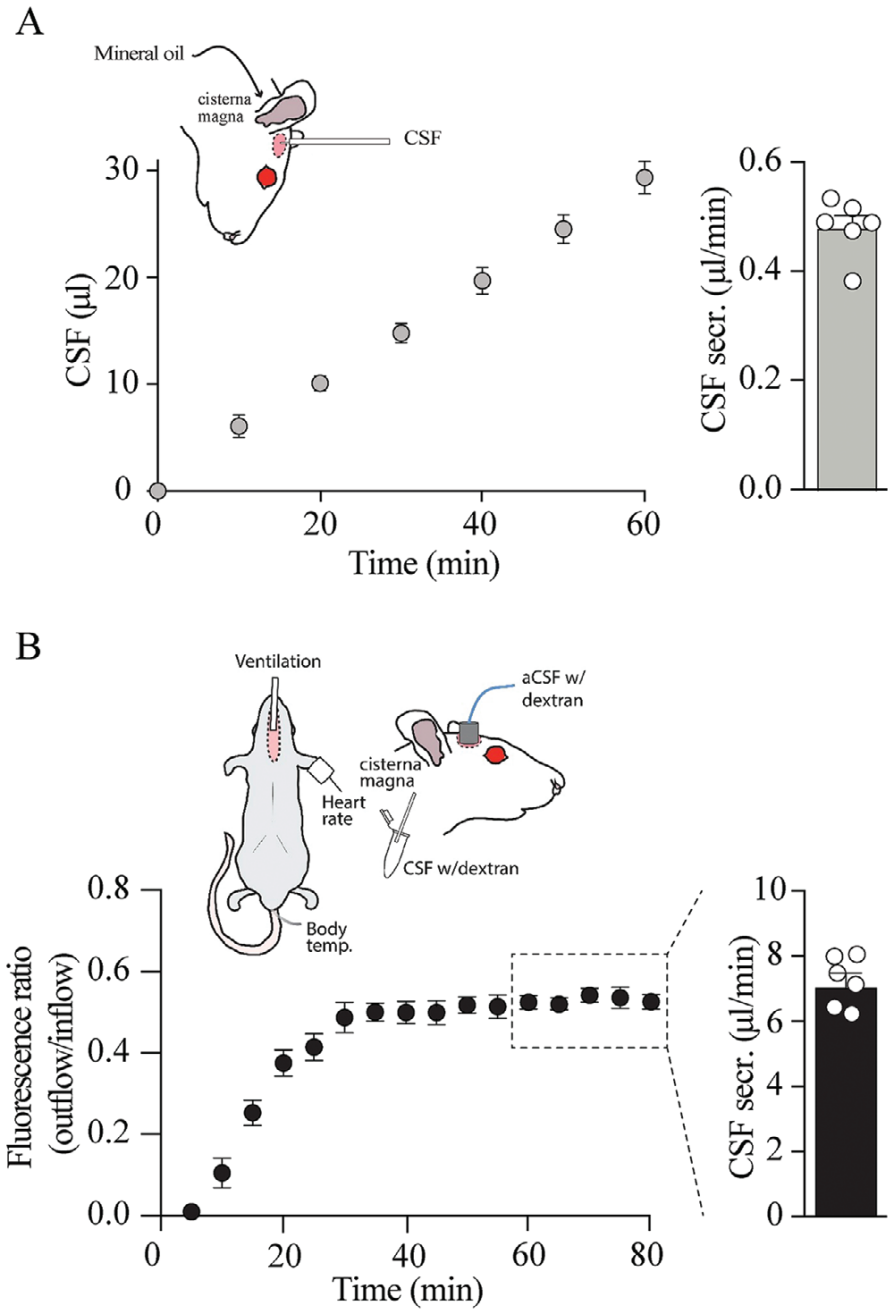
Different methods yield different CSF secretion rates. A) Schematic of the experimental setup employing the “direct method” with summarized CSF collection as a function of time and the resulting rate of CSF secretion (n = 6). B) Schematic of the experimental setup employing ventriculo‐cisternal perfusion with the summarized time course of the fluorescence ratio (outflow/inflow) of dextran and resulting CSF secretion rate at time points 60–80 min (indicated by the dashed box, n = 6).

The alternative manner of CSF secretion measurement, the “direct method,” relies on blockage of the access route between the 3^rd^ and 4^th^ ventricles; the Aqueduct of Sylvius, with microinjection of mineral oil into the 4^th^ ventricle.^[^ ^23^ ^]^ The newly secreted CSF may subsequently exit through a brain cannula inserted into the lateral ventricle, and the rate of CSF passage through the cannula then employed to compute the CSF secretion rate.^[^ ^23^ ^]^ This procedure currently does not allow mechanical ventilation of the rat, given the required head position. The CSF secretion rate obtained in this manner in the 9‐week old Sprague–Dawley rats here employed arrived at 0.48 ± 0.02 µL min^−1^, n = 6, **Figure** **5**A. This CSF secretion rate is significantly lower than that obtained in a parallel batch‐matched series of ventriculo‐cisternal perfusion assay in mechanically ventilated rats (CSF secretion rates of 7.20 ± 0.34 µL min^−1^, n = 6, *P* < 0.001, Figure 5B). Notably, two rats died prior to completion of the non‐ventilated “direct method” and were omitted from the quantitation of the CSF secretion rates.

### 2.4 The Different CSF Secretion Methodologies Accurately Reflect Changes in CSF Secretion Rates

**Figure 6:**
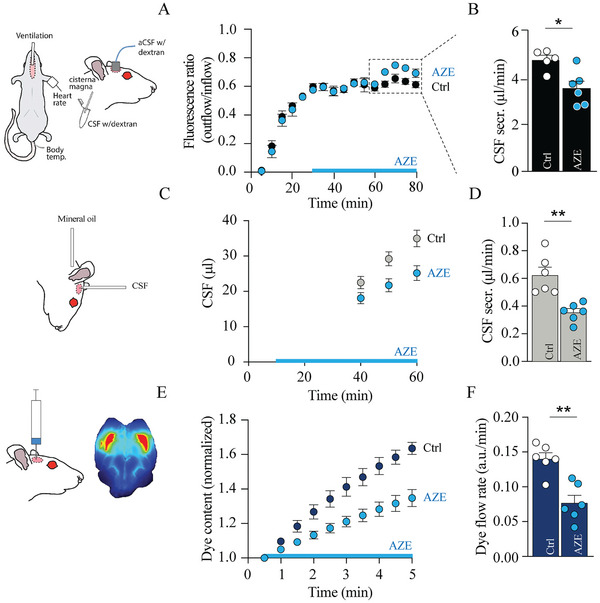
Different CSF secretion assays accurately reflect relative changes in CSF secretion rates. A) Schematic of the experimental setup employing ventriculo‐cisternal perfusion with summarized time courses of the fluorescence ratio (outflow/inflow) of dextran in control (Ctrl) and acetazolamide‐treated (AZE) rats, n = 5–6. The blue bar indicates AZE/vehicle administration window. B) Summarized CSF secretion rates at time points 60–80 min (indicated by the dashed box in A) in control (Ctrl) and acetazolamide‐treated (AZE) rats, n = 5–6. C) Schematic of the experimental setup employing the “direct method” with summarized time CSF collection as a function of time in control (Ctrl) and acetazolamide‐treated (AZE) rats, n = 6. The blue bar indicates AZE/vehicle administration window. D) Summarized CSF secretion rate in control (Ctrl) and acetazolamide‐treated (AZE) rats as determined by the slopes of the volume‐time relationships in C (n = 6 of each). E) Schematic of the experimental setup employing live imaging of the caudal flow of a fluorescent dye injected into the lateral ventricle as a proxy for CSF secretion with summarized time courses of dye flow in control (Ctrl) and acetazolamide‐treated animals (AZE), n = 6 of each. The blue bar indicates AZE/vehicle administration window. F) Quantification of the dye intensity (flow rate) determined from linear regression in E over a 5 min time window. Statistical significance determined by Student's *t*‐test. ^*^ *P* < 0.05, ^**^ *P* < 0.01.

Although the absolute value of the CSF secretion rate appears to depend on the methodology employed, most research in the CSF secretion field centers on *changes* in CSF secretion rates with various inhibitors, genetic modifications, age, sex, pathology, etc.^[^ ^7^, ^11^, ^23^, ^24^, ^25^, ^31^ ^]^ We therefore determined the reduction in CSF secretion rate with a well‐established inhibitor of CSF secretion; acetazolamide (AZE) across three different techniques of measuring CSF secretion. AZE inhibits carbonic anhydrases throughout the mammalian body^[^ ^34^ ^]^ and lowers the CSF secretion rate in all tested species.^[^ ^11^, ^23^, ^35^, ^36^, ^37^, ^38^, ^39^, ^40^, ^41^, ^42^, ^43^, ^44^, ^45^ ^]^ Its specific action on the choroid plexus carbonic anhydrases leads to reduced CSF secretion and ensuing lowering of the ICP in experimental rats 30 min post‐delivery.^[^ ^11^ ^]^ To resolve the effect of AZE on CSF secretion when measured with the traditional methods, we treated the rats with AZE (intraperitoneal administration, i.p.) during the experimental series, while ensuring that the quantification of the AZE‐mediated reduction in CSF secretion was quantified 30 min post‐injection. AZE reduced the CSF secretion rate when recorded in ventilated rats with the ventriculo‐cisternal perfusion assay (5.20 ± 0.47 µL min^−1^ in control rats vs 3.55 ± 0.30 µL min^−1^ in AZE‐treated rats; 32%, n = 5–6, P < 0.05, **Figure** **6**A,B). With the “direct method”, AZE reduced the CSF secretion rate to a similar extent to what was observed with the ventriculo‐cisternal perfusion assay (0.62 ± 0.06 µL min^−1^ in control rats vs 0.35 ± 0.03 µL min^−1^ in AZE‐treated rats; 43%, n = 6 of each, P < 0.01, Figure 6C,D). Lastly, live imaging (LI‐COR) of the caudal flow of a fluorescent dye injected into the lateral ventricle as a proxy for CSF secretion,^[^ ^25^, ^28^ ^]^ also demonstrated a similar AZE‐mediated reduction in CSF secretion (0.14 ± 0.01 a.u. min^−1^ in control rats to 0.08 ± 0.01 a.u. min^−1^ in AZE‐treated rats; 42%, n = 6, P < 0.01, Figure 6E,F). Relative changes in CSF secretion can thus be accurately determined with all three experimental methods to measure CSF secretion rates.

## 3 Discussion

Here, we present a novel method to quantify CSF secretion in unanesthetized and freely moving rats, and reveal that anesthesia, but not lack of mechanical ventilation, lowers the CSF secretion rate in young adult rats. Different methodologies provide different CSF secretion rates, although *changes* in CSF secretion rate are reliably reflected with the different experimental approaches. With the requirement of invasive experimental methodology to quantify CSF secretion rates under these various parameters, ethical considerations prevent these approaches to be carried out in human subjects, which thus augment the value of rodent experiments such as those employed in the present study.

The absolute CSF secretion rates have proven elusive to determine accurately, as there is no generally accepted method that reliably provides absolute values of CSF secretion rates in humans or experimental animals. Over the years, it has been proposed that the volume CSF secreted per choroid plexus mass across the tested species arrives at ≈0.5 µL min^−1^ mg^−1^ choroid plexus,^[^ ^21^, ^30^, ^46^ ^]^ which proposes an estimated CSF secretion rate of 2.5 µL min^−1^ in young adult rats harboring ≈5 mg choroid plexus tissue.^[^ ^26^ ^]^ This value represents an intermediate value between that obtained with the ventriculo‐cisternal perfusion assay (≈6 µL min^−1^ in rats, in this study and^[^ ^11^ ^]^ and that obtained with the “direct method” (≈0.5 µL min^−1^ in this study and^[^ ^23^ ^]^). Corresponding published values in mice are ≈0.7^[^ ^25^ ^]^ and ≈0.08 µL min^−1^,^[^ ^24^ ^]^ respectively, thus demonstrating that the CSF secretion rate is approximately one magnitude larger with one assay over the other, whether obtained in rats or mice. Notably, the CSF secretion rate in rats here obtained with the direct method (0.48 ± 0.02 µL min^−1^) was slightly lower than those reported by Karimy and colleagues (0.74 ± 0.05 µL min^−1^).^[^ ^23^ ^]^ This discrepancy may, in part, originate from our higher doses of xyl/ket required for deep anesthesia (omission of all foot reflexes in the experimental animals), which aligns with the present findings of xyl/ket‐dependent reduction in CSF secretion compared to awake animals and the inverse relation between CSF secretion and a number of ketamine re‐dosings. Each of these methods has their advantages and disadvantages, with the “direct method” i) excluding the contribution from the large 4^th^ choroid plexus, which in animals is predicted to produce half of the CSF,^[^ ^47^, ^48^ ^]^ ii) preventing mechanical ventilation (due to the required head angle), iii) potential blockage‐induced CSF leakage into the brain parenchyma, iv) not readily allowing intraventricular delivery of inhibitors/modulators (as exemplified with the 50–100‐fold higher inhibitor concentrations required than those employed with the ventriculo‐cisternal perfusion assay and the LI‐COR live measurement of CSF flow).^[^ ^7^, ^11^, ^23^ ^]^ However, its swift procedure and technical ease is beneficial. The ventriculo‐cisternal perfusion assay i) is rather lengthy and technically challenging, ii) may^[^ ^23^ ^]^ or may not (this study) depend on perfusion speed, iii) may allow CSF indicator diffusion into the brain parenchyma with the omission of the cisternal puncture that is inherent to the ventriculo‐perfusion assay,^[^ ^18^, ^24^, ^49^ ^]^ although not detectable in our hands following a completed experiment,^[^ ^11^ ^]^ and iv) requires perfusion of equiosmolar, gas‐equilibrated, and pre‐heated HCO_3_^−^‐based aCSF (as here employed) to exclude confounding elements entering with the ventricular perfusion. The ventriculo‐cisternal perfusion assay readily allows intraventricular delivery of CSF secretion inhibitors/modulators with the perfusion solution.^[^ ^11^, ^25^ ^]^

The majority of CSF secretion research focuses on *relative changes* in the rate of CSF secretion with various inhibitors/activators, age groups, sex, pathologies, etc.,^[^ ^7^, ^11^, ^23^, ^24^, ^25^, ^31^ ^]^ in which the *absolute* CSF secretion rate is not important. We here demonstrate that AZE, which is a generally accepted inhibitor of CSF secretion in humans and animals,^[^ ^11^, ^23^, ^35^, ^36^, ^37^, ^38^, ^39^, ^40^, ^41^, ^42^, ^43^, ^44^, ^45^ ^]^ inhibits the CSF secretion rate with ≈40% in all three experimental assays; the ventriculo‐cisternal perfusion assay, the “direct method,” and when monitoring CSF flow with live imaging. Notably, AZE was here administered i.p., which provides the same inhibitory action as when given intravenously, intracerebroventricularly (i.c.v.), and per orally in awake, as well as anesthetized, animals^[^ ^11^ ^]^ and overcomes the inherent challenge with i.c.v. delivery with the “direct method.” Inhibition of several transport proteins involved in CSF secretion (the Na^+^/K^+^‐ATPase, the Na^+^,K^+^,2Cl^−^‐cotransporter (NKCC1), and the Na^+^‐driven HCO_3_^−^ cotransporter (NBCe2)^[^ ^25^, ^26^ ^]^ or of the regulatory transient receptor potential vanilloid 4 (TRPV4) ion channel^[^ ^11^ ^]^ has previously been demonstrated to provide the same reduction of CSF secretion, whether determined with the ventriculo‐cisternal perfusion assay or the LI‐COR‐based live imaging of CSF flow,^[^ ^11^, ^25^ ^]^ thus cementing reliable determination of relative changes in CSF secretion rates with any of the three experimental methods.

To circumvent the changes in physiological parameters and CSF dynamics that may come about with the various anesthesia paradigms employed, we established a novel method, based on the principle of the ventriculo‐cisternal perfusion assay, to determine CSF secretion in awake and freely moving rats. All experiments were conducted 16 h post‐implantation of brain access ports, and the rats were only briefly exposed to isoflurane while connecting the tubing to their harness. Determination of CSF secretion rates was obtained with the rats roaming the test cage and also under isoflurane anesthesia to obtain a paired experimental scenario conducted at the same time of day in the same rats with the same tubing. All rats displayed 30% higher CSF secretion rates during their awake state, with a reversal of the order of awake‐isoflurane (with 90 min intervals) in half of the experimental rats to avoid time‐dependent confounders. The isoflurane‐induced reduction in CSF secretion was mimicked with xyl/ket anesthesia in parallel experiments with batch‐matched rats, which aligns with an earlier demonstration of similar CSF secretion rates in mice with either of these anesthesia paradigms.^[^ ^25^ ^]^ In contrast, an earlier study revealed an increased CSF secretion rate with xyl/ket or isoflurane anesthesia compared to awake, but head‐fixed, mice,^[^ ^24^ ^]^ the difference of which may arise, in part, from the experiments being conducted 30 min post‐surgical preparation for the “direct method,”^[^ ^18^, ^24^ ^]^ as opposed to 16 h post‐implantation in the present study. Of note, though, previously demonstrated xyl/ket‐induced reduction of murine CSF *clearance* ^[^ ^50^, ^51^ ^]^ aligns with the here observed xyl/ket‐induced reduction in rat CSF *secretion*. Such match may explain the similar ICPs measured in anesthetized versus unanesthesized rat.^[^ ^11^ ^]^

It remains unresolved what mechanisms underlie the anesthesia‐induced changes in CSF secretion rates. One may speculate that the increased levels of rodent plasma corticosterone,^[^ ^52^ ^]^ altered plasma catecholamine levels,^[^ ^53^ ^]^ and/or the reduced blood pressure and heart rate observed in rats with both anesthesia paradigms^[^ ^26^ ^]^ may directly or indirectly affect the CSF secretion rate, as could the diminished vasomotor reactivity detected in ketamine‐treated patients.^[^ ^54^ ^]^ Some of these physiological parameters may be normalized with mechanical ventilation of the animal during the experimental procedure and could, as such, rescue the anesthesia‐induced reduction of the CSF secretion rate.^[^ ^55^ ^]^ The present data illustrate that neither rats nor mice require mechanical ventilation during the ventriculo‐cisternal perfusion assay to sustain their CSF secretion in the tested time window. However, non‐ventilated animals had a tendency to die during the experimental procedure (3/9 rats died prior to completion of the experiment versus none in the ventilated group, and 2/5 mice died toward completion of the experiment vs none in the ventilated group). Both rats and mice had lower blood oxygenation, which for rats was accompanied by a higher heart rate, but otherwise undisturbed electrolyte and metabolite blood levels. The similar CSF secretion rates suggest that the oxygenation levels obtained from natural breathing during the experiments suffice for proper CSF secretion in the employed age groups. Notably, in a parallel study with aged rats, these required immediate mechanical ventilation upon xyl/ket anesthesia induction to survive even the initiation of the experimental procedure (*unpublished data*). The requirement for mechanical ventilation during an experimental procedure may thus depend on rodent age.

## 4 Limitations

As with all in vivo experimentation of this invasive nature, the present study is limited by potential confounding elements that could affect our results. Here, we test the effect of anesthesia, of mechanical ventilation, of ketamine (re‐)dosing, of infusion rates, and of different experimental techniques. Nevertheless, there may be a range of other limitations that ought to be tested and quantified prior to settling on a given experimental design. For example, a limitation common to all three experimental methodologies is the requirement for a cranial burr hole to allow access to the ventricular system during data acquisition. It remains unresolved what impact such a surgical approach will have on brain homeostasis in general, and – more specifically – on the CSF secretion rate. A zeroing of the ICP, as created by a burr hole, could, in itself, alter the CSF secretion rate. However, with the current technical limitations regarding methodology to quantify the CSF secretion rate, it simply remains unknown whether a burr hole will alter the CSF secretion rates, both in the short term, as here employed, and in potential applications with long‐term use of burr holes. In addition, the experimental technique here employed to determine CSF secretion rates in awake rats merely quantifies the effect of anesthesia on the CSF secretion rate and therefore does not reveal potential anesthesia‐induced changes in CSF flow, glymphatic exchange, lymphatic activity, or CSF drainage, which represent other pivotal parameters in brain fluid dynamics likely to be affected by anesthesia. Future research may, in addition, address the potential anesthesia‐induced changes in CSF content of biomarkers, such as amyloid‐beta and tau.

## 5 Conclusion

In conclusion, we here – with a novel method – demonstrate that the CSF secretion is higher in un‐anesthetized and freely moving rats than in their anesthetized counterparts, although the absolute obtained values of CSF secretion rate depend on the experimental method employed. Importantly, all three tested methods accurately reflect a percentage reduction in CSF secretion obtained with a conventional pharmacological inhibitor of this physiological process. It follows that experimental designs aimed at semi‐quantification of relative differences in CSF secretion rates may employ either of these procedures, at least with systemic delivery of inhibitors, which may facilitate the establishment of CSF secretion experimentation in more laboratories.

## 6 Experimental Section

### Animals

Experiments were conducted in 9–10 weeks old male Sprague Dawley rats (Janvier Labs) and 10–11 weeks old male wild‐type mice (C57BL/6J, Janvier) that were housed in a temperature‐controlled room with a 12 h:12 h light‐dark cycle (6 am to 6 pm) with free access to a standard rodent pellet diet and tap water. All animal experimental work conformed to the legislations for animal protection and care in the European Community Council Directive (EU Directive 2010/63/EU) and approved by the Danish Animal Experiments Inspectorate (license no. 2021‐15‐0201‐00867 and 2018‐15‐0201‐01595). Animals were randomly allocated to each treatment group.

### Solutions and Chemicals

The ventriculo‐cisternal perfusion assay was conducted with HCO_3_^−^‐buffered a CSF(containing in mM) 120 NaCl, 2.5 KCl, 2.5 CaCl_2_, 1.3 MgSO_4_, 1 NaH_2_PO_4_, 10 glucose, 25 NaHCO_3_, gas‐equilibrated with 95% O_2_/5% CO_2_. In experiments where the solution could not be equilibrated with 95% O_2_/5% CO_2_, during the experimental procedure (LI‐COR live CSF imaging), the solution was instead buffered by HEPES (HEPES‐aCSF; (in mM) 120 NaCl, 2.5 KCl, 2.5 CaCl_2_, 1.3 MgSO_4_, 1 NaH_2_PO_4_, 10 glucose, 17 Na‐HEPES, adjusted to pH 7.4 with NaOH at the relevant working temperature). Acetazolamide (AZE, A6011, Sigma‐Aldrich) was dissolved in 5 N NaOH to a 700 mg mL^−1^ stock solution, which was diluted in 0.9% NaCl to a working concentration of 20 mg mL^−1^, pH 8.4 with an equiosmolar NaCl solution, pH 8.4, serving as control (vehicle) solution. The AZE solution was administered i.p. (5 mL kg^−1^ rat arriving at 100 mg k^−1^g,^[^ ^11^ ^]^ equivalent to a single human clinical dose)^[^ ^56^ ^]^ and timed to be administered exactly 30 min prior to quantification of the CSF secretion rate.

### Anesthesia and Physiological Parameter Monitoring

For ventriculo‐cisternal perfusion, the “direct method” and LI‐COR live imaging, the experimental animals were anesthetized with an i.p. injection with xyl/ket (ScanVet, 6 mg mL^−1^ xylazine + 60 mg mL^−1^ ketamine; 0.17 mL per 100 g body weight for rats and 1 mg mL^−1^ xylazine + 10 mg mL^−1^ ketamine; 0.1 mL per 10 g body weight for mice) in sterile water (pre‐heated to 37 °C). Note, that the doses of anesthesia reported in^[^ ^23^ ^]^ did not suffice for deep anesthesia in our hands. For ventriculo‐cisternal perfusion, the animals were re‐dosed with half ketamine dose, as per detection of foot reflex, as required to sustain anesthesia. Survival surgery (placement of brain access ports) was performed under aseptic conditions on rats anesthetized with isoflurane (Attane vet, 1000 mg g^−1^ isoflurane, ScanVet), using 5% isoflurane (mixed with 1.8 l min^−1^ air/0.2 l min^−1^ O_2_) to induce anesthesia in an induction chamber, and 2–2.5% to maintain anesthesia through a facemask, which was gradually decreased to 1‐1.5% during the surgical procedure. The body temperature of the anesthetized rats was maintained at 37 °C by a homeothermic monitoring system (Harvard Apparatus). Mechanical ventilation was included for some anesthetic protocols to ensure stable respiratory partial pressure of carbon dioxide (pCO_2_) and arterial oxygen saturation (pO_2_). A surgical tracheotomy was performed, and the ventilation was controlled by the VentElite system (Harvard Apparatus) by 0.9 l min^−1^ humidified air mixed with 0.1 l min^−1^ O_2_ adjusted with ≈3 mL/breath, 80 breaths min^−1^, 10% sigh, and a Positive End‐Expiratory Pressure (PEEP) at 2 cm H_2_O for a ≈ 400 g rat, and ≈150 µL/breath, 150 breaths min^−1^, 10% sigh, and a PEEP at 2 cm H_2_O for a  ≈ 25 g mouse. The ventilation settings were optimized for each animal using a capnograph (Type 340, Harvard Apparatus) and a pulse oximeter (MouseOx® Plus, Starr Life Sciences) after system calibration with respiratory pCO_2_ (4.5–5.0 kPa), pO_2_ (13.3–17.3 kPa), and O_2_ (98.8–99.4%) (ABL90, Radiometer). Arterial blood was sampled from the femoral artery of the rats at the termination of the ventriculo‐cisternal perfusion experimental series (both in ventilated and non‐ventilated animals; t = 120 min), and blood pCO_2_, pO_2_, K^+^, Na^+^, Cl^−^, glucose, lactate, and HCO_3_^−^ levels were quantified with an ABL90 FLEX blood gas analyzer (Radiometer, Copenhagen, Denmark).

### CSF Secretion Determination with Ventriculo‐Cisternal Perfusion

Anesthetized and non‐ventilated experimental animals had an infusion cannula (Brain infusion kit 2, Alzet, with 1 mm adjustment spacers) stereotactically placed in the right lateral ventricle through a 0.5 mm burr hole drilled into the skull. For the rats, the coordinates were 1.3 mm posterior to bregma, 1.8 mm lateral to the midline, and 4 mm ventral into the brain and the alzet glued to the skull with Superglue (Pelikan). For the mice, the coordinates were 0.5 mm posterior to bregma, 1.0 mm lateral to the midline, and 2.5 mm ventral into the brain, and the alzet secured to the skull with dental resin cement (Panavia SA, Kuraray Noritake Dental Inc.). Pre‐heated (37 °C, SF‐28, Warner Instruments) HCO_3_^−^‐aCSF containing 0.5 mg mL^−1^ TRITC‐dextran (tetramethylrhodamine isothiocyanate‐dextran, MW  =  150000; T1287, Sigma–Aldrich) was infused at 6 or 9 µL min^−1^ in rats and 0.7‐0.8 µl min^−1^ in mice (controlled by a peristaltic pump) and CSF was sampled from the cisterna magna at 5 min intervals with a glass capillary (30–0067, Harvard Apparatus pulled by a Brown Micropipette puller, Model P‐97, Sutter Instruments) placed at a 5° angle (in rats: 7.5 mm distal to the occipital bone and 1.5 mm lateral to the muscle‐midline). The fluorescent dextran content of sampled CSF was measured in triplicates on a microplate photometer (545 nm, SynergyTM Neo2 Multi‐mode Microplate Reader; BioTek Instruments), and the CSF secretion rate was calculated from the equation: V_p_ = r_i_ x (C_i_ – C_o_)/C_o_ where V_p_ = CSF secretion rate (µL min^−1^), r_i_ = infusion rate (µL min^−1^), C_i_ = fluorescence of inflow solution, C_o_ = fluorescence of outflow solution^[^ ^57^ ^]^ at time points 55 – 80 min after initiation of the ventricular infusion.

### CSF Secretion in Awake Rats

Experimental rats were anesthetized with isoflurane, the head and neck shaved and initially sterilized with 0.5% chlorhexidine (Medic). Pre‐operative analgesia was provided with subcutaneous (s.c.) injections of carprofen (5 mg k^−1^g, Norodyl Vet, Norbrook) and buprenorphine (0.05 mg k^−1^g, Tamgesic, Indivior) and local (s.c.) delivery of 400 µL of a mix of lidocaine (5 mg mL^−1^)/bupivacaine (2.5 mg mL^−1^). The rats were placed in a stereotactic frame, had their skull exposed, and a burr hole drilled, as above, for placement of an alzet. Two additional drill holes were made for anchor screws (0‐80×1/16, Bilaney Consultants GmbH) that were secured with resin cement (Panavia SA Cement Universal, Kuraray Noritake GmbH). The rat was repositioned to promote a 120° neck angle, and the atlanto‐occipital membrane was carefully exposed. The membrane was punctured with a 23‐gauge needle with subsequent insertion of a tube (PE#50,*BD Intramedic*), custom modified by burning one end to create a circular plate but retaining an open lumen, into the cisterna magna and anchored with tissue adhesives (BRAU9381104, B. Braun) and silicone tubing (228‐0701 VWR). The incision site was sutured (non‐absorbent suture, Ethicon) and the rat was provided with a harness (VAHD115AB, Instech) with connector inflow/outflow ports. The two tubes (one connected to the alzet for aCSF infusion (as described for the ventriculo‐cisternal perfusion assay) and one inserted into the cisterna magna for sampling outflow) were connected to inflow/outflow ports in the harness. After 16 h recovery, the rats were briefly anesthetized with isoflurane to test the ventricular flow by connecting an injector (lateral ventricle injector attached to aCSF filled syringe, VAH6M‐50, Instech) to the harness. The harness was connected to a tether (VADH115T, Instech), and the rat was placed in the test cage (MTANK/WF), containing bedding from their home cage, with a lever arm (MCLA) and swivel (375/D/22), all from Instech, to freely acclimatize for 10 min. The CSF secretion quantification was initiated by infusion of dextran‐containing HCO_3_^−^‐aCSF infusion into the lateral ventricle (8 µl min^−1^, controlled by a peristaltic pump) and simultaneous cisternal CSF sampling (by a peristaltic pump), and analyzed at time points 55–75 min. Half of the rats were subsequently anesthetized with isoflurane, and their CSF secretion rate was determined, while the other half underwent the procedures in the opposite order to ensure no time‐dependent experimental confounder, both with 90 min break between the two experimental paradigms to ensure full recovery from the anesthesia.

### CSF Secretion Determination with the “Direct Method”

Rats were anesthetized and mounted in a stereotactic frame and a 1.3 mm burr hole was drilled 0.8 mm posterior and 1.7 mm lateral to bregma. The hind end of the rat was then elevated ≈2 cm and the head rotated on the ear‐bars (90°), nose downward, and held in this vertical position using an in‐house made snout holder pressing against the dorsum of the nose. The suboccipital muscles were dissected from the occipital bones and exposed from the atlanto‐occipital ligament to expose the cisterna magna. The atlanto‐occipital ligament was partly resected, and the cisterna magna was punctured. A 23‐gauge flexible catheter (PE‐20) was loaded with pre‐warmed (37 °C), sterile, molecular grade mineral oil (100 µL, Sigma–Aldrich) and gently advanced 5 mm through the foramen of Magendie into the 4^th^ ventricle where the oil was infused to occlude the Aqueduct of Sylvius. Following this occlusion, the lateral ventricle was cannulated through a4 mm burr hole with a glass capillary (OD 1 mm; ID 0.8 mm; length 30 cm; VitroCom). The first capillary often contained brain matter and was withdrawn and replaced by reintroducing a new capillary into the ventricle. The capillary was gradually filled with CSF exiting from the lateral ventricle, and the distance that the front of the CSF column traveled within the capillary measured at 5 min intervals was measured and the volume of newly formed CSF was calculated as: volume = π × radius^2^ × distance. The rate of CSF formation (µL min^−1^) was calculated as the slope of the volume‐time relationship.^[^ ^23^ ^]^

### CSF Secretion Determination with LI‐COR Live Imaging

Anesthetized rats were placed in a stereotactic frame. The cranium and upper neck muscles were exposed, and a burr hole drilled (same coordinates as ventriculo‐cisternal perfusion) into which a Hamilton syringe (RN 0.40, G27, a20, Agntho's) containing 15 µL aCSF with carboxylate dye (10 µM; IRDye 800CW, P/N 929–08972, LI‐COR Biosciences) was inserted 4 mm into the lateral ventricle. The solution was injected at 1.5 µL s^−1^ and image acquisition was initiated 1 min after carboxylate injection (and 30 min post‐AZE administration) and continued for 5 min with 30 s intervals using a Pearl Trilogy Small Animal Imaging System (LI‐COR) (800 nm channel, 85 µm resolution). The anesthetized rats were secured during imaging in a custom‐made tooth holder to stabilize their head position. The fluorescence signal was determined in a region of interest (ROI) placed at skull landmark lambda as a function of time, and quantified relative to the initial fluorescence intensity obtained within the first micrograph captured at time point 30 s. A white field image of the rat head was captured at the end of imaging prior to visualizing the lateral ventricles of the isolated brain hemispheres to verify bilateral carboxylate staining. Data analyses were performed in Image Studio 5.2 (LI‐COR Biosciences – GmbH, Nebraska, US).

### Data Presentation and Statistics

All data are presented as mean ± SEM. Statistical significance was tested with Student's *t*‐test and one‐way ANOVA with Tukey's multiple *post‐hoc* test as indicated in the figure legends. *P* values < 0.05 were considered statistically significant. The number of experiments (*n*) corresponds to measurements in independent animals.

## Conflict of Interest

The authors declare no conflict of interest.

## Author Contributions

T.L.T‐B., B.L.E. and A.B.S. contributed equally to this work. Conception and design of research: A.B.S., B.L.E., T.L.T‐B. and N.M.; Data acquisition: A.B.S., B.L.E., T.L.T‐B., S.D.L., D.B.J., J.H.W.; Data analysis, A.B.S., B.L.E., T.L.T‐B., S.D.L.; Drafting of manuscript: T.L.T‐B., A.B.S. and N.M. All authors revised and approved the final version of the manuscript.

## Supporting information



Supporting Information

## Data Availability

Data from the study is available from the corresponding author upon reasonable request.
