# Supplementary material for: The Cerebrospinal Fluid Secretion Rate Increases in Awake and Freely Moving Rats but Differs With Experimental Methodology
Source: Adv Sci (Weinh). 2025 Mar 12;12(17):2412469. doi: 10.1002/advs.202412469 (PMC12061303; doi:10.1002/advs.202412469)
Supplement: Supplementary file 1 — Supporting Information [file ADVS-12-2412469-s001.pdf]

ADVANCED SCIENCE
Open Access

Supporting Information

for *Adv. Sci.*, DOI 10.1002/advs.202412469

The Cerebrospinal Fluid Secretion Rate Increases in Awake and Freely Moving Rats but Differs With Experimental Methodology

Trine L. Toft-Bertelsen, Beatriche L. Edelbo, Annette B. Steffensen, Sara D. Lolansen, Jonathan H. Wardman, Dennis B. Jensen and Nanna MacAulay**

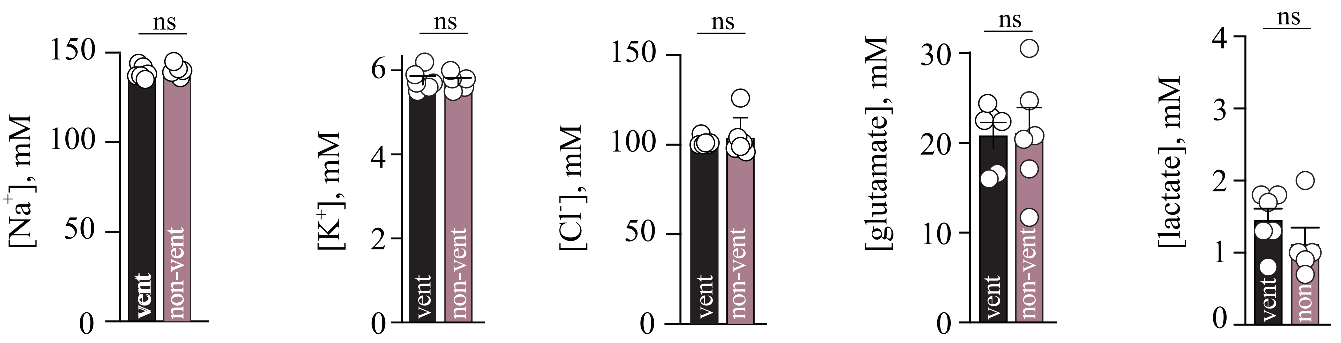

Figure S1. Blood electrolytes in ventilated and non-ventilated rats. Plasma [Na$^+$], [K $^+$], [Cl $^-$],[glucose] and [lactate] in ventilated (black) and non-ventilated (purple) rats. n = 5-6 of each. Statistical significance determined by one-way ANOVA with Tukey's multiple post-hoc test.
